# Association Between Fatty Acids Profile and Cerebral Blood Flow: An Exploratory fNIRS Study on Children with and without ADHD

**DOI:** 10.3390/nu11102414

**Published:** 2019-10-10

**Authors:** Silvia Grazioli, Alessandro Crippa, Maddalena Mauri, Caterina Piazza, Andrea Bacchetta, Antonio Salandi, Sara Trabattoni, Carlo Agostoni, Massimo Molteni, Maria Nobile

**Affiliations:** 1 Scientific Institute, IRCCS E. Medea, 23842, Bosisio Parini, Italy; alessandro.crippa@lanostrafamiglia.it (A.C.); maddalena.mauri@lanostrafamiglia.it (M.M.); caterina.piazza@lanostrafamiglia.it (C.P.); andrea.bacchetta@lanostrafamiglia.it (A.B.); antonio.salandi@lanostrafamiglia.it (A.S.); sara.trabattoni@lanostrafamiglia.it (S.T.); massimo.molteni@lanostrafamiglia.it (M.M.); maria.nobile@lanostrafamiglia.it (M.N.); 2 PhD Program in Neuroscience, School of Medicine and Surgery, University of Milano-Bicocca, 20126 Milan, Italy; 3 Pediatric Intermediate Care Unit, Fondazione IRCCS Ca’ Granda—Ospedale Maggiore Policlinico, 20122 Milan, Italy; carlo.agostoni@unimi.it; 4 DISSCO Department of Clinical Sciences and Community Health, University of Milan, 20122 Milan, Italy; 5 SIGENP (Italian Society of Pediatric Gastroenterology, Hepatology, and Nutrition), via Libero Temolo 4 (Torre U8), 20126 Milan, Italy

**Keywords:** polyunsaturated fatty acids, NIRS, cerebral blood flow, ADHD, biomarker, attention, rehabilitation

## Abstract

Polyunsaturated fatty acids (PUFAs) biostatus has been proposed as possible attention deficit hyperactivity disorder (ADHD) diagnosis biomarker. The present exploratory study aimed to investigate the association between PUFAs biostatus and cerebral cortex metabolism measured by functional Near Infrared Spectroscopy (fNIRS) in a sample of children with and without ADHD. 24 children with ADHD and 22 typically developing (TD) peers, aged 8–14, were recruited. Linoleic, arachidonic, docosahexaenoic and eicosapentaenoic acids levels were evaluated in whole blood. All children underwent fNIRS while performing an n-back working memory task. Between groups comparisons revealed lower levels of arachidonic acid in children with ADHD and stronger NIRS signal in TD participants, especially when completing more difficult tasks. Correlations conducted between fNIRS activation and PUFA biostatus revealed several associations between hemodynamic changes in the frontoparietal regions and fatty acids profile across participants. This result was also confirmed by the multiple hierarchical regression analyses that remarked an inverse effect of eicosapentaenoic acid levels on oxyhemoglobin values in right frontoparietal region. Such preliminary findings, if confirmed, would suggest that PUFAs could play a role in atypical neurodevelopment.

## 1. Introduction

Attention deficit hyperactivity disorder (ADHD), which affects approximately 7.2% of children worldwide, is the most common childhood neurodevelopmental disorder [1]. ADHD is a complex and multifactorial condition. Its core symptoms reflect a lack of self-regulating behavior, cognition and emotional responses, as well as difficulties in paying attention, excessive motor activity and high impulsivity levels [2].

ADHD manifestations are heterogeneous and impact cognition and behavior through a multifaceted aetiopathogenesis that involves biological, psychosocial, and environmental aspects.

Given this complexity, an objective diagnosis biomarker has not been established. Conventionally, the ADHD diagnostic process relies on subjective criteria, such as clinical interviews, observations, and rating scales. Insight into the biological markers associated with typical and atypical development could offer clinicians more reliable tools for ADHD assessment and the opportunity to implement targeted treatment plans [3].

Taking these premises into account, there has been growing interest in recent years in the role of nutrition in the development, treatment, and prevention of neurodevelopmental disorders. In particular, attention has been focused on the role of polyunsaturated fatty acids (PUFAs) as possible biological markers in ADHD (see, for example, [4,5,6]), suggested also by the relevant role for these biological components in normal brain functionality [7].

PUFAs represent an indispensable component of lipids that cover a relevant role in human diet and biological functions such as provision of energy, functionality of cell membranes and tissue metabolism [8]. The simplest lipids are fatty acids, which contain a hydrophilic carboxyl group, a hydrocarbon chain and a methyl group. Fatty acids carbon chain can be saturated (with no presence of double bonds) or unsaturated (with one or more double bonds). PUFAs fall into the unsaturated group, and they can be divided into two classes: omega-3 (n-3) and omega-6 (n-6) fatty acids (FAs), which are distinguished by the location of their double bonds in the carbon chain [9].

Cis-linoleic acid (LA 18:2, n-6) and α-linoleic acid (ALA 18:3, n-3) are considered “essential” fatty acids because mammals cannot biosynthesize them. They are primarily plant-derived and exclusively available from diet. Mammals metabolize LA and ALA in long-chain PUFAs: n-6 PUFAs (e.g., arachidonic acid - AA 20:4, n-6) derived from LA and n-3 PUFAs (e.g., docosahexaenoic acid-DHA 22:6 n-3-and its precursor eicosapentaenoic acid-EPA 20:5 n-3) derived from ALA [10,11]. PUFAs are relevant components of cellular membranes, phospholipids, and precursors of eicosanoids, which influence neuronal development and functioning [12]. DHA and AA are the brain’s major lipid constituents and, in different roles, are involved in cell growth, neural signaling, and gene expression [7,8]. Long-chain FAs have pro- and anti-inflammatory properties. Generally, AA and DHA have opposite effects on synaptic transduction and inflammation [7]. AA and its derived eicosanoids tend to exhibit pro-inflammatory activity in various cell types and disorders [10,11,12]. Nevertheless, AA also plays an important role in brain cell division and signaling [13,14].

EPA, DHA and their derived eicosanoids, on the other hand, are powerful regulators of biological process, with anti-inflammatory properties [10,12]. The main natural dietary source for EPA and DHA is fish oil.

DHA represents the 10–20% of the fatty acids in mammalian grey matter. It is important for neuronal cell membrane integrity and for the signaling system because it influences various neurotransmitter pathways, including the cholinergic system, which is known to play a key role in memory and learning [7,15]. Therefore, DHA plays a crucial role in the cellular structure of the cerebral cortex, especially within the frontal lobe [16]. The physiology of this brain region, which is involved in executive functions and behavioral and emotional regulation, has been often reported as altered in individuals diagnosed with ADHD [17].

On the other hand, EPA-derived eicosanoids regulate the immune and endocrine systems and cardiovascular functions [7].

N-3 deficiency and high n-6/n-3 ratios are indicators of inflammation and have been linked with various mental disorders, including ADHD [10,16]. In addition, evidence from preclinical studies indicates that n-3 deficiency may be associated with impaired attention and learning ability [18,19].

Within the last decade, an increasing number of studies has evaluated the association between PUFA intake or biostatus and human brain structure and function using various neuroimaging methods. To date, studies involving healthy subjects indicate that increased dietary fish intake and higher blood levels of DHA and EPA are usually associated with increased grey matter volume and greater activity-dependent cerebral blood flow [7]. The relationship between n-3 and cognitive performance might be partly mediated by grey matter volume [7].

Near infrared spectroscopy (NIRS) is a viable approach with which to obtain information about cerebral cortex metabolism in a non-invasive manner and at relatively low cost. NIRS is an optical technique that uses light at specific wavelengths to detect changes in oxygenated and deoxygenated hemoglobin (HbO and HbR, respectively) concentrations over time [20]. NIRS has been successfully used to detect activations of the frontal, parietal, and temporal cortices during neuropsychological tasks [21,22,23]. It is therefore an optimal choice when studying children with neurodevelopmental disorders [24,25].

Hamazaki-Fujita and colleagues [26] used NIRS with healthy adults and found that increased EPA and DHA levels were associated with increased tissue oxygenation indices in the prefrontal cortex while completing an arithmetic task. A similar association was not found in another cross-sectional study on healthy male adults performing a working memory task [27]. However, the participants’ fish consumption was associated with a greater increase in HbO in the left dorsolateral prefrontal cortex during the task.

A clinical trial conducted on healthy adult volunteers evaluated the effect of DHA and EPA supplementation on cortical hemodynamics measured using functional NIRS (fNIRS) during a cognitive task that elicited prefrontal cortex activation [28]. The study revealed a significant effect of DHA, but not EPA, in increasing cerebral HbO in prefrontal cortex when compared to the placebo condition. In a second double-blind, placebo-controlled trial [29], the authors evaluated the effect of DHA-rich fish oil at a dose of 1 or 2 g on cerebral blood flow measured using NIRS. In total, 65 healthy adult participants performed nine computerized cognitive tasks. Both doses of DHA, when compared with the placebo, were associated with an increased concentration of HbO and total Hb, as well as a dose-response effect on total Hb concentrations, during cognitive tasks, but no effect on behavioral performances.

Although PUFA supplementation is one of the most popular non-pharmacological treatments for ADHD [30], few studies have evaluated the association between FAs, cerebral activation, and cognition in children with ADHD.

To our knowledge, only Bos and colleagues [30] have investigated the effect of n-3 PUFAs on ADHD in a randomized placebo-controlled trial of young boys with and without ADHD diagnoses using functional magnetic resonance imaging during a go-no go paradigm. n-3 supplementation improved inattention symptoms in both groups; however, no effect was found on brain activity or task performance after supplementation.

Based on previous considerations, the goal of the present study was to investigate the association between cerebral hemodynamic activation measured using fNIRS and blood PUFA levels in children with ADHD and typically developing (TD) peers. To elicit the cortical hemodynamic responses, we used an n-back working memory task with blocks of increasing difficulty [31]. The same task was used by Crippa, Salvatore and colleagues [32] to successfully differentiate patients with ADHD from TD children by means of their cortical hemodynamic responses measured with fNIRS. To the best of our knowledge, no studies have examined the association between PUFA biostatus and task-dependent cortical activity in children with and without ADHD.

## 2. Materials and Methods 

The present study is a cross-sectional, observational study of children with and without ADHD that investigates the association between PUFA biostatus and brain hemodynamic responses to a cognitive task with differing workloads as measured with fNIRS. Our research is part of a placebo-controlled double-blind intervention trial (June 2012–October 2014) that explored the efficacy of DHA supplementation in children with ADHD (“The Effects of DHA on Attention Deficit and Hyperactivity Disorder - DADA Study”). The trial was registered at ClinicalTrials.gov as NCT01796262 and was approved by our institute’s ethics committee in accordance with the Declaration of Helsinki (1967) and its later amendments. Written informed consent and assent were obtained from all caregivers and participants.

### 2.1. Participants

#### 2.1.1. Children with ADHD

As a part of a larger project [12], 24 children with ADHD aged 8 to 14 were recruited from our institute’s child psychopathology unit over a 22-month period. All children had been previously diagnosed with ADHD according to Diagnostic and Statistical Manual of Mental Disorders criteria (4th ed., text rev.; DSM-IV-TR; APA, 2000) by a child neuropsychiatrist with expertise in ADHD. Moreover, a child psychologist experienced in the diagnosis of ADHD (AC) independently confirmed the diagnosis through direct clinical observation and the administration of the semi-structured interview Development and Well-Being Assessment (DAWBA) [33]. According to the clinical assessment, 16.7% of the children met the criteria for the ADHD inattentive subtype, 33.3% fulfilled criteria for the hyperactive–impulsive subtype, and 50% had the combined subtype. 

#### 2.1.2. Typically Developing Children

Twenty-one TD children living in the same areas as the children with ADHD were recruited as a control group. The research team excluded possible neuropsychiatric diagnoses in control subjects, using the DAWBA parent diagnostic interview. The genders and ages of TD children were matched to those of the clinical sample. Children in the control group completed the vocabulary and block design subtests of the Wechsler Intelligence Scale for Children-III (WISC-III) [34] to estimate their Full-Scale Intelligence Quotient (FSIQ). This measure was used to match the clinical and control groups [35].

Regarding the whole research sample, only participants with FSIQ scores or estimated FSIQ scores higher than 80 were included. Moreover, all children were drug-naïve and did not consume omega-3 or omega-6 supplements during the 3 months prior to recruitment. Exclusion criteria included a history of seizures, other psychiatric or neurological disorders, and diagnosed genetic disorders. All participants were Caucasian and had normal or corrected-to-normal vision.

### 2.2. Measures

Participants were assessed at our institute’s child psychopathology unit. Each child’s weight and the blood pressure were measured. Familial socioeconomic status was coded according to the Hollingshead scale for parental employment [36].

#### 2.2.1. Cognitive and Clinical-Behavioral Profile

Four subtests from the Amsterdam Neuropsychological Tasks (ANT) [37] were administered to evaluate executive function domains: baseline speed, focused attention 4 letters, shifting attentional set–visual, and sustained attention. Furthermore, participants’ parents completed the Conners’ Parent Rating Scale (CPRS-R) [38] and ADHD rating scale IV - Parent Version-Investigator (ADHD-RS) [39] to assess ADHD behavior severity. More detailed information regarding cognitive and behavioral domain measures is available in [40]. 

#### 2.2.2. Fatty Acids Profile

After a minimum 1-hour fast, biological samples were obtained from all children by collecting drops of blood using an automatic lancing device that punctured the participant’s fingertip; a strip of paper was used to collect each sample. FAs profile was evaluated in whole blood, which is more easily obtainable than other blood components, such as red blood cells and plasma. Thus, we measured the status of long- and short-chain circulating FAs in relation to dietary habits [41]. All blood samples were preserved at 4 °C until they were analyzed through transmethylation for gas chromatography analysis using a well-validated protocol [42]. We detected FAs from 14 to 24 carbons, and their values were expressed as a percentage of total FAs. In the present work, we report single-FA data for the main n-3 and n-6 FAs: LA, AA, EPA, and DHA.

#### 2.2.3. Stimulation Protocol

**Figure 1:**
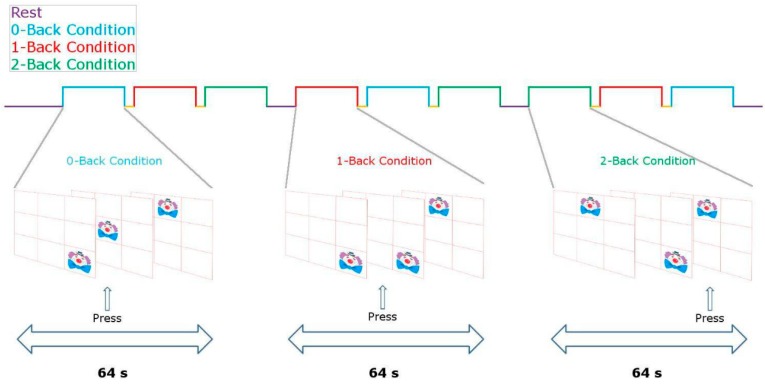
Task design.

During fNIRS recording, each participant completed a computerized protocol (approximately 15 min long) developed with the Presentation® software (Neurobehavioral Systems Inc., Denver, CO, USA. The stimulation protocol was a variant of the visuospatial n-back working memory task (n-back VSWM) developed by Cui and colleagues [31,32]. The paradigm consisted of a rest condition and three tasks with increasing difficulty [0-back, 1-back, and 2-back] (see Figure 1). During rest conditions, children passively viewed a white cross on a black screen. In the three task conditions, a clown’s face was displayed on a location of a 3 × 3 matrix. In the 0-back task, children were instructed to respond only when the clown’s face was presented in the center of the screen. In the 1-back task, children were required to respond if the stimulus remained in the same position as in the previous trial. In the 2-back task, participants had to respond if the stimulus recurred in the same location, as in the two previous trials. Each experimental epoch included 32 stimuli that lasted 0.5 seconds each, with a 1.5-second inter-stimulus interval.

**Figure 2:**
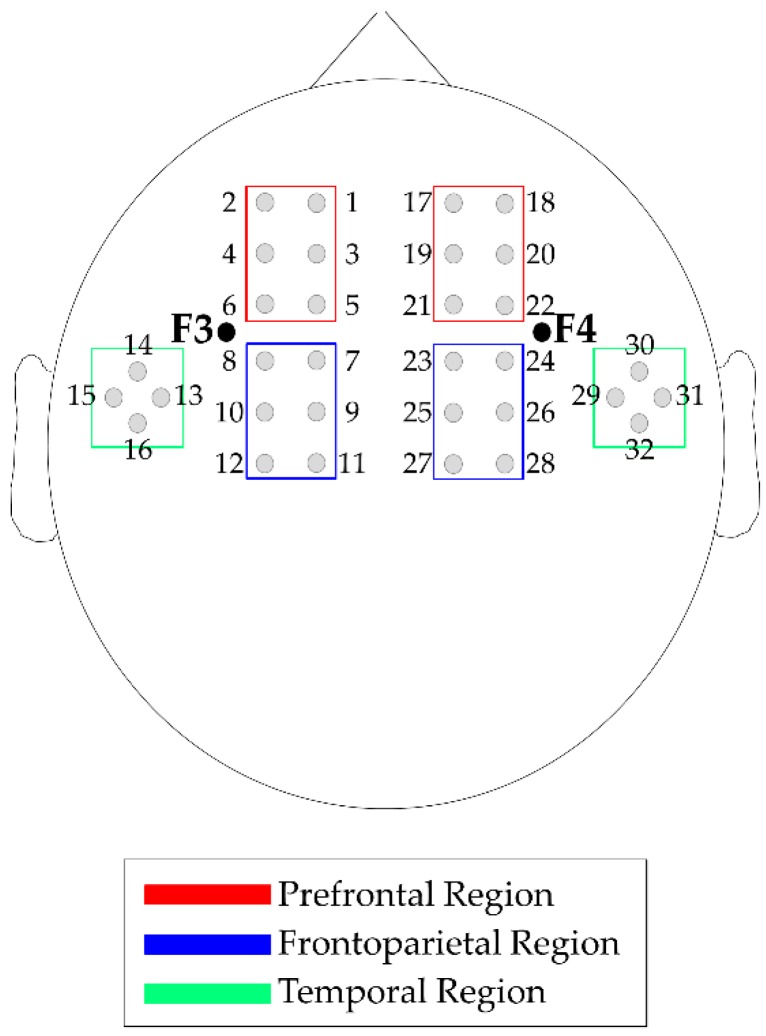
fNIRS channels and region of interest (ROI) map. Note: Sources and detectors are localized on a 10–20 system. Left-prefrontal ROI: Channels 1–6. Left-frontoparietal ROI: Channels 7–12. Right-prefrontal ROI: Channels 17–22. Right-frontoparietal ROI: Channels 23-28. Temporal areas were not considered in the analyses.

fNIRS data acquisition, optode localization and data preprocessing. We acquired fNIRS data with a commercial continuous-wave NIRS device (DYNOT Compact 9-32, NIRx, Berlin, Germany). An elastic cap of the proper head size with 32 channels was used. Specifically, 8 light sources and 24 light detectors were placed on the bilateral frontotemporal areas centered at F3 and F4, according to the International 10–20 system [43]. The source–detector distance was 2.7 cm (Figure 2). Recordings were performed at two wavelengths, 760 nm and 830 nm, to measure both HbO and HbR concentration changes after data conversion using the modified Beer-Lambert Law [44]. NIRS data preprocessing was performed using the Homer2 v2.3 software [45,46].

#### 2.2.4. Neurophysiological Profile

The preprocessed fNIRS time series and prior to fNIRS analysis, were standardized applying at each data point (p0) of the nine task blocks the following formula:

p1 = (p0 − m_3s_)/s_3s_

where m_3s_ and s_3s_ are the mean and the standard deviation of the 3 seconds before the block’s beginning [32,47]. Then, time point concentration data were averaged accordingly to the different task conditions. Specifically, four task conditions were identified: 0-back (0B), 1-back (1B), 2-back (2B) and overall task (“Task”), i.e., the three-condition considered together. To evaluate the channels more involved in the task execution, the possible activations of each channels in respect to the baseline were identified by performing channel-wise Wilcoxon tests on the control group, between the “Task” and a time window of the baseline “Rest” (from 25 seconds to 5 seconds before the first task section) for HbO and HbR. Based on the Wilcoxon test results, bilateral temporal ROIs (channels 13–16 and channels 29–32) were excluded from further analysis because fewer than half of the channels were able to detect significant Hb changes. Finally, HbO and HbR data were averaged in four ROIs to increase signal-to-noise ratio; each was composed of six contingent channels: left-prefrontal (l-PF: channels 1–6), left-frontoparietal (l-FP: channels 7–12), right-prefrontal (r-PF: channels 17–22) and right-frontoparietal (r-FP: channels 23–28) (see Figure 2).

Statistical analyses were conducted using the SPSS statistical software package (IBM Corp. Released 2012. IBM SPSS Statistics for Windows, Version 21.0. Armonk, NY: IBM Corp.).

Data were visually and statistically inspected to check whether the variables were normally distributed and to address assumptions of linearity, independence of observations and homogeneity of error variance. Fisher’s exact test, Mann-Whitney, and independent-samples *t* test analyses (according to variables distribution) were conducted to examine between-group differences for demographic, clinical, cognitive, behavioral (n-back task performance) measures, and blood FAs levels.

#### 2.2.5. Neurophysiological Profile

Mann-Whitney or two-tailed independent-samples *t* test analyses (depending on the distributional nature of the data) were computed to examine the between-groups difference of activation in each task condition for HbO and HbR in the four ROIs.

#### 2.2.6. ADHD Severity-Neurophysiological Profile Association

To investigate possible associations between cortical hemodynamics and ADHD severity in the clinical sample, Spearman correlations were conducted between fNIRS activation significantly different between-groups and clinical scales for ADHD severity (ADHD index from CPRS-S and “*Total*” measure from ADHD-RS). Since this study was exploratory, no correction was applied for family-wise error rate. However, confidence intervals (CIs) of 95% were calculated for Spearman’s Rho using the bootstrapping method (1000 bootstrap resamples) to indicate the likely size of the effect [48].

#### 2.2.7. Fatty Acids-Neurophysiological Profile Association

Relationships between fatty acid composition (LA, AA, EPA, and DHA) and HbO and HbR activation in each task condition in the four ROIs were analyzed using Spearman correlations. After verifying the assumptions and controlling for the effects of age, socioeconomic status, and total IQ, we conducted multiple hierarchical regression analyses to estimate HbO or HbR changes to varying blood FAs biostatuses, with FA level as an independent variable and brain hemodynamic activation as a dependent variable [49].

## 3. Results

Sample demographic characteristics and FAs biostatus are reported in Table 1 and Table 2, respectively.

Demographic characteristics were balanced between groups (all *p* > 0.05).

With respect to participants’ blood FA profiles, children with ADHD showed lower levels of AA compared to typically developing peers. No additional significant difference was found regarding the FA profile.

The groups’ cognitive and clinical characteristics are illustrated in the Supplementary Materials (Table S1). Task performances during fNIRS are depicted in Table S2.

Several significant between-group differences were found in cognitive variables (ANT, Table S1). Children with ADHD committed more inhibition errors (visual set-shifting) and showed more difficulties with sustained attention, as highlighted by an increased variability in reaction times (sustained attention).

Moreover, as expected, participants with ADHD manifested significantly higher values in all clinical measures of ADHD (ADHD rating scale and Conners’ parents rating scales, Table S1).

Lastly, a statistical assessment of data revealed two outliers in the ADHD group in terms of number of errors during n-back task (2 standard deviations from the next highest score). Thus, the two participants’ data were excluded from all further analysis. We found similar performances across participants for the n-back task, except for slower responses to stimuli in the 0B condition in children with ADHD. No other significant differences were found in any task condition.

### 3.1. Neurophysiological Profile

**Figure 3:**
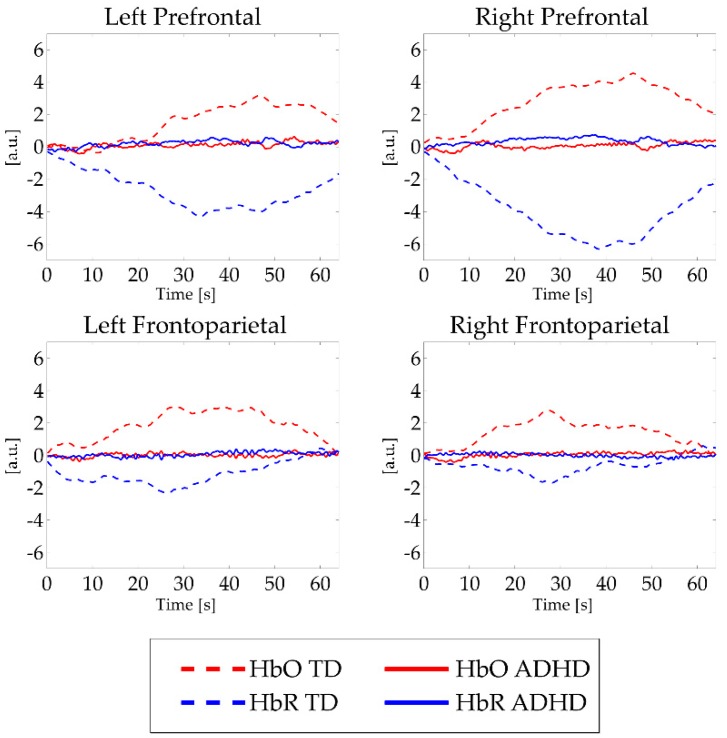
Mean fNIRS activations in all task conditions. Note: a.u. = Arbitrary units; s = seconds. Red lines indicate HbO signal, blue lines depict HbR signal. Hatch pattern represents activations from typically developing children, continuous lines illustrate activations in children with ADHD. For significant between-group differences, see Table S3a, S3b.

The results of between-groups differences for mean HbO and HbR activation in each ROI and task condition are summarized in Table S3a and S3b in the Supplementary Materials. Absolute values of HbO and HbR data showed weaker activation in subjects with ADHD compared to TD children in each area and task condition. These findings reached statistical significance level in several ROIs and workloads, as depicted in Figure 3.

### 3.2. ADHD Severity-Neurophysiological Profile Association. 

With respect to the association between hemodynamic activations that differentiate the clinical group from the TD group (see Table S3a, S3b) and ADHD severity scores, our results showed a marginally significant positive correlation (Spearman’s rho = 0.501, *p* < 0.05) between HbO values in right frontoparietal ROI in the 2-back condition and Conners’ ADHD index. No additional significant correlation was found between HbO or HbR signals and ADHD clinical scores.

### 3.3. Fatty Acids-Neurophysiological Profile Association

Table S4a, S4b in the Supplementary Materials section show the Spearman correlation results. With respect to HbO values, the Spearman coefficients revealed a significant negative correlation between left frontoparietal values in the 0-back condition, all task conditions and EPA blood levels. In the right frontoparietal ROI, significant negative correlations were found between HbO values in the 0-back condition and both AA and EPA.

Regarding HbR levels, significant correlation coefficients were found in the right frontoparietal ROI: positive associations were found between HbR in the 0-back condition and EPA biostatus and HbR in the 2-back condition and AA levels; a negative correlation was found between HbR in the 1-back condition and DHA value. Lastly, a significant negative correlation coefficient was found for left prefrontal HbR in the 1-back condition and DHA.

Table 3 shows multiple hierarchical regression results.

Our regression model with AA and EPA fatty acids as independent variables and HbO concentration in right frontoparietal ROI, in 0-back condition, as dependent variable (controlling for age, socioeconomic status and total IQ), was statistically significant. Our model explained 26.8% of variance (adjusted R^2^ = 0.268, F = 3.786, *p* = 0.008). Specifically, EPA blood levels had a significant negative effect on HbO concentration (β = –0.494, *t* = –3.280, *p* = 0.015), and, as such, explained approximately 24.6% variance of HbO concentration in right frontoparietal ROI, in 0-back condition.

## 4. Discussion

In the last decades, an increasing body of research has drawn considerable interest to the possible impact of dietetic factors on ADHD symptomatology as playing a potential role in the pathophysiology of the condition and, therefore, as a possible coadjutant non-pharmacological intervention to pharmacological and psychological treatment [11].

Given these premises, our study was aimed at evaluating the association between blood PUFA biostatus and cerebral hemodynamics as measured by fNIRS in a sample of Italian children with and without ADHD. To the best of our knowledge, no studies have examined the association between PUFA biostatus and fNIRS data in a sample with the above-mentioned characteristics.

To this end, we recruited two groups of school-aged children, one composed of children with a clinical diagnosis of ADHD and one composed of TD children; all were of average IQ.

As a preliminary step, we compared individual PUFA biostatus and task-related cortical activations between the two groups of children, observing lower levels of AA in children with ADHD than in TD children. As expected, this result is consistent with our preliminary findings in children with ADHD in an Italian setting [40]. The importance of AA in humans and other mammals is indicated by the variety of functions that this FA performs in a range of metabolic events in spite of relatively constant levels in body pools [13,14]. Blood AA deficiencies in children with ADHD and other learning disorders have been reported previously [50,51]. However, the reason for the specific neurocognitive effect of lower blood AA levels in children with atypical development, especially with ADHD, remains unclear.

In regard to the changes in the oxygenated and deoxygenated hemoglobin concentrations measured by fNIRS during performance of n-back working memory tasks, we found different activation patterns in children with ADHD than in their TD peers (see Figure 3). The results indicated a stronger increase in HbO and HbR concentrations in healthy participants compared to the ADHD group. The present results suggest that ADHD children, in light of n-back task performances comparable to healthy controls, show weaker cortical activation when completing more difficult tasks, especially when the tasks require more complex stimuli retention (1-and 2-back conditions). We found no differences in HbO or HbR concentrations between ADHD and TD participants in the 0B condition, which is traditionally ascribed to indexes of selective attention rather than working memory [51]. These findings are consistent with a previous fNIRS study by Ehlis and colleagues that used similar cognitive tasks in patients with ADHD [52]. Again, in a functional magnetic resonance imaging study, Kobel and colleagues [51] also found significantly higher activation in bilateral frontal and parietal areas related to the increasing difficulty of n-back tasks in children with typical development compared to peers with ADHD. Dorso-lateral areas cover a relevant role in manipulation of information (in our case, 1B and 2B conditions) [52]. Conversely, ventral areas activity has been consistently found in association with detection of transient stimuli (0B condition, in our task) [52]. Thus, the lack of between-group differences in lower executive functions load could be possibly reconducted to our NIRS probe configuration that enables the identification of dorso-lateral areas hemodynamic activity rather than ventral cortices metabolism. However, these peculiarities in cerebral blood dynamics seem to be limited to the physiological level and not significantly correlated to ADHD severity, as evaluated by parents through CPRS and ADHD rating scales. This observation extends our previous findings [31] suggesting that the characterization of ADHD functioning could be enriched by broader biological measures, including PUFA biostatus and cortical hemodynamic data. In the actual clinical practice, ADHD is diagnosed on the basis of symptoms as judged by clinicians and by means of qualitative measures. We believe that the integration of information from different sources and levels of analysis (biological, behavioral, cognitive, and neurophysiological) could lead to a more comprehensive description of children with ADHD, supporting the clinical practice of diagnosing ADHD.

With respect to the main goal of this study—to investigate the association between blood PUFA biostatus and cortical hemodynamics—we found, regardless of diagnosis, a few puzzling findings when exploring PUFA percentages and changes in the concentration of oxygenated and deoxygenated hemoglobin (see Table 3). Most correlations between FAs and fNIRS signals were found for hemodynamic changes in the frontoparietal regions. Moreover, these associations seem more evident for HbO and HbR values recorded in the 0-back condition. This weakly significant trend was confirmed by the regression model results. When controlling for participants’ sociodemographic characteristics, such as age, intellectual functioning and socioeconomic status, we found an inverse effect of EPA blood levels on HbO values measured over the right frontoparietal region only in the 0-back condition. This baseline task condition requires concentration and selective attention from the participants rather than more complex and demanding cognitive processes, such as working memory. Paradoxically, Hamazaki-Fujita and colleagues [26] also found a significant association, though in the opposite direction, between EPA and change in tissue oxygenation index estimated by fNIRS in healthy adults performing a simple arithmetic task that did not require complex mental manipulation of the information. A supplementation study [28] reported no effect of EPA-rich fish oil on cerebral blood flow measured using fNIRS in adults. Nevertheless, this significant degree of heterogeneity in findings could be explained by the 250–300-fold lower EPA concentrations in brain compared to the DHA concentration, raising concerns on its consistency [53].

In light of this consideration, we were surprised to find no significant association between DHA, which derives from EPA and cortical hemodynamic changes after having controlled for the participants’ demographics. As also described in the Introduction, DHA is the brain’s major lipid constituent and plays a critical role in maintaining membrane integrity and fluidity and influencing inter-cell communications. Even though several previous studies that used various imaging techniques suggested a direct effect of DHA on cortical blood flow with regard to the effect of DHA supplementation [28,29,54] or as simple association with DHA status [53,55], results in literature are controversial [9]. The causes of this discrepancy could be various, as the present work differed from the previous studies in several important methodological aspects, such as the participants’ ages, the nature of the cognitive task and the fNIRS parameters used to evaluate changes in hemoglobin concentrations. In addition, it is possible that the association between DHA (as well as AA) and cortical hemodynamics would be better explained by additional latent variables not included in our regression model (i.e., genetics of enzymatic steps, grey matter volume, and dietary intakes of PUFAs). These variables should be taken into account in future extensions of the present study. We highlight that associations between DHA levels and brain functionality might depend on manifold factors [56,57], that could have intervened also in the conflicting statistical effects found in our work. For instance, we did not control for dietary sources of PUFAs, aspect that could have contribute to the discrepancy between EPA and DHA results.

The present study has relevant limitations. The novelty of our approach did not allow us to contextualize the results in a broader research area; in fact, to the best of our knowledge, there is no previous study addressing PUFA and brain cortex metabolism in a developmental-aged sample of children with and without ADHD. Previous fNIRS studies conducted with healthy adults gave inconsistent results [25,27], which are hardly comparable with ours. Therefore, this work was limited by its small sample size mainly due to its exploratory nature, and the statistical testing was not adjusted for multiple comparisons (many of the experimental measures were intercorrelated). Indeed, even though some correlations between PUFA and fNIRS data were found, the rho values were generally low. Greater sample size is needed to generalize these preliminary results. In addition, there was also a disproportionate gender distribution across participants. Future studies addressing gender differences could be fruitful. Finally, it is biologically plausible that the inverse relation found between brain metabolism and PUFA biostatus could be influenced by a number of latent confounding factors not considered in this work, such as children’s genetic characterization of PUFA metabolism, total brain volumes and dietary intakes of PUFAs, as underlined also elsewhere.

## 5. Conclusions

For the first time in the literature, the present exploratory study suggests a possible association between blood FA composition and cerebral hemodynamics in a sample of children with ADHD and TD peers. Such preliminary findings should be now considered as a relevant area of research and addressed in samples with sufficient size to disentangle possible mediating effects of genetics, brain structure or volume, nutrition and ADHD subtypes, and allowing for family-wise error rate correction as appropriately required. If confirmed, these results would suggest that dietary components, such as FAs, could play a role in atypical neurobehavioral development.

## Figures and Tables

**Table 1:** Sample demographic characteristics.

	ADHD	TD	Value	*p*
*N*	24	21	-	-
F:M	0:24	1:20	1.169 ^a^	0.467
Age	11.5 ± 1.5	11.3 ± 1.8	−0.485 ^b^	0.630
IQ	101.8 ± 11.1	110 ± 20	1.683 ^b^	0.103
SES	54 ± 20.2	56 ± 18.7	0.344 ^b^	0.734

Note. ADHD = Children with ADHD; Controls = Typically developing children; F = Females; M = Males; IQ = Intelligence quotient; SES = Socioeconomic status. ^a^ Fisher’s Exact Test; ^b^ Student’s *t* test.

**Table 2:** Blood fatty acids analysis data.

	ADHD	TD	Value ^a^	*p*
LA	22.80 ± 2.34	22.63 ± 2.47	−0.242	0.810
AA	9.34 ± 1.19	10.07 ± 0.94	2.264	0.029
EPA	0.98 ± 0.56	1.13 ± 0.46	0.963	0.341
DHA	1.75 ± 0.49	1.92 ± 0.54	1.107	0.274

Note. ADHD = Children with ADHD; TD = Typically developing children; LA = linoleic acid; AA = arachidonic acid; EPA = eicosapentaenoic acid; DHA = docosahexaenoic acid. Contrast in bold is significant at alpha = 0.05. ^a^ Student’s *t* test value.

**Table 3:** Multiple hierarchical regression results with arachidonic acid (AA) and eicosapentaenoic acid (EPA) as independent variables, age, socioeconomic status (SES) and intelligence quotient (IQ) as covariates, and HbO in right frontoparietal ROI in 0B condition as dependent variable.

AA and EPA → rFP HbO, 0B	Regression Coefficients		
	***β* [Bootstrap c.i.]**	*t*	*p*
Age	−0.323 [−0.936; 0.051]	−1.976	0.055
IQ	0.042 [−0.050; 0.057]	0.259	0.810
SES	0.216 [−0.006; 0.061]	1.351	0.094
AA	−0.030 [−0.860; 0.562]	−0.208	0.840
**EPA**	**−0.494 [−4.678; −0.813]**	**−3.280**	**0.015**
	Model summary		
	Adjusted R^2^	F	*p*
Age, IQ, SES	0.070	1.955	0.139
AA and EPA	**0.268**	**3.786**	**0.008**

Note. **→** = AA and EPA are the independent variables of the multiple hierarchical regression; 0B = 0 Back task condition; AA = Arachidonic acid; *β =* Regression beta coefficient; c.i. = confidence intervals; EPA = Eicosapentaenoic acid; F = F value; HbO = Oxyhemoglobin; IQ = Intelligence quotient; rFP = Right frontoparietal area; ROI = Region of interest; SES = Socioeconomic status; *t* = *t* value; *p* = probability value; R^2^ = R-squared (coefficient of determination). Values in bold are significant according to *p* value and bootstrap confidence intervals. Lower and upper limits of 95% confidence intervals from bootstrapping methodology with 1000 resamples iteration are reported in square brackets.

## Supplementary Materials

The following are available online at https://www.mdpi.com/2072-6643/11/10/2414/s1, Table S1: Cognitive and clinical measures (means ± standard deviations) in participants groups.; Table S2: N-back task performance (means ± standard deviations).; Table S3a: HbO (a.u.) mean activations in each ROI and task condition in both groups. Table S3b: HbR (a.u.) mean activations in each ROI and task condition in both groups; Table S4a: Spearman’s rho values for correlations between HbO and blood fatty acid measures in the whole sample; Table S4b: Spearman coefficients values for correlations between HbR and blood fatty acid measures in the whole sample.
