# Supplementary material for: Association Between Fatty Acids Profile and Cerebral Blood Flow: An Exploratory fNIRS Study on Children with and without ADHD
Source: Nutrients. 2019 Oct 10;11(10):2414. doi: 10.3390/nu11102414 (PMC6836039; doi:10.3390/nu11102414)
Supplement: Supplementary file 1 [file nutrients-11-02414-s001.pdf]

Table S1. Cognitive and clinical measures (means ± standard deviations) in participants groups.

	ADHD (n = 24)	Controls (n = 21)	Value	*p*		ADHD (n = 24)	Controls (n = 21)	Value	*p*
**ANT—Baseline speed**					**ANT—Sustained attention date**				
RT (ms)	328.54 ± 64.93	317.38 ± 45.57	250.000[b]	.964	*SD*	3.39 ± 1.46	2.41 ± 1.20	-2.406[a]	**.020**
SD of RT	118.71 ± 83.83	87.05 ± 22.84	192.500[b]	.176	Misses	29.88 ± 20.14	19.57 ± 18.75	-1.768[a]	.084
**ANT—Focused attention 4 letters**					False alarms	17.04 ± 9.36	19.52 ± 18.65	233.000[b]	.665
RT correct responses (ms)	926.66 ± 275.60	963.52 ± 355.97	.391[a]	.698	Coefficient of variation	.22 ± .07	.17 ± .05	-3.073[a]	**.004**
SD of correct responses RT	324.65 ± 181.63	337.94 ± 189.53	231.000[b]	.633	**ADHD rating scale**				
Misses	2.63 ± 2.37	2.14 ± 2.13	-.713[a]	.480	Hyperactivity–Impulsivity scale	14.79 ± 1.93	1.86 ± 5.90	-10.141[a]	**<.001**

False alarms relevant non-target	.58 ± .83	.52 ± .75	-.251[a]	.803	Inattention scale	15.67 ± 6.23	2.52 ± 2.98	-9.201[a]	**<.001**
False alarms irrelevant target	1.87 ± 3.78	.86 ± 1.28	190.000[b]	.137	Total	30.46 ± 10.75	4.38 ± 4.46	-10.862[a]	**<.001**
**ANT—Visual set-shifting**					**Conners' parents rating scales**				
RT inhibition (ms)	296.50 ± 206.76	409.76 ± 286.15	191.000[b]	.165	ADHD index	72.79 ± 11.16	42.52 ± 5.95	-11.546[a]	**<.001**
RT flexibility (ms)	644.71 ± 298.09	792.83 ± 374.11	202.000[b]	.255	CGI: restless–impulsive	69.29 ± 11.80	41.95 ± 6.80	-9.661[a]	**<.001**
Number of errors inhibition	8.21 ± 7.80	3.52 ± 4.32	-2.533[a]	**.016**	CGI: emotional lability	60.88 ± 15.64	43.81 ± 15.64	-5.076[a]	**<.001**
Number of errors flexibility	18.04 ± 11.97	14.90 ± 14.60	184.500[b]	.124	CGI: total	66.79 ± 16.29	41.57 ± 6.21	-7.024[a]	**<.001**
					DSM-IV: inattentive	69.00 ± 17.02	44.05 ± 6.99	-6.577[a]	**<.001**
					DSM-IV: hyperactive–impulsive	68.04 ± 16.01	40.95 ± 3.63	-8.054[a]	**<.001**
					DSM-IV: total	71.30 ± 15.01	41.71 ± 5.20	-8.887[a]	**<.001**

Note. ADHD = Children with ADHD; Controls = Typically developing children; RT = Mean reaction time; *SD* = Standard deviation; ANT = Amsterdam Neuropsychological Task.
Contrast in bold is significant at alpha = .05.
[a]Student's *t* test value. [b]Mann-Whitney test value.

Table S2. N-back task performance (means ± standard deviations).

	ADHD	Controls	Value	*p*
**0 Back condition**				
Mean RT	4691.18 ± 898.17	4038.57 ± 731.50	-2.605[a]	**.013**
SD	1345.64 ± 538.40	1069.48 ± 485.80	-1.763[a]	.085
Number of errors	.50 ± .74	.19 ± .40	-1.714[a]	.096
Misses	.23 ± .43	.14 ± .48	203.00[b]	.288
**1 Back condition**				
Mean RT	5901.27 ± 1226.84	5432.48 ± 1446.34	-1.148[a]	.258
SD	2206.86 ± 888.57	1828.24 ± 910.97	-1.38[a]	.175
Number of errors	1.95 ± 1.29	1.76 ± .94	-.557[a]	.581
Misses	1.68 ± 2.42	.86 ± 1.77	184.50[b]	.206
**2 Back condition**				
Mean RT	6149.41 ± 1471.20	5417.24 ± 1780.17	-1.473[a]	.148
SD	2830.23 ± 1315.95	2550.05 ± 1316.20	-.698[a]	.489
Number of errors	2.05 ± 3.55	.57 ± .75	169.50[b]	.108
Misses	7.50 ± 4.32	5.24 ± 3.92	-1.795[a]	.080
**Total**				
Mean RT	5580.64 ± 980.49	4962.71 ± 1182.77	-1.869[a]	.069
SD	2127.59 ± 438.55	1815.90 ± 718.59	-1.707[a]	.097
Number of errors	4.50 ± 4.60	2.52 ± 1.60	167.50[b]	.115
Misses	9.41 ± 5.80	6.24 ± 4.55	-1.989[a]	.053

Note. ADHD = Children with ADHD; Controls = Typically developing children; RT = Reaction time; *SD* = Standard deviation. Contrast in bold is significant at alpha = .05. [a] Student's *t* test value. [b]Mann-Whitney test value.

Table S3a. HbO (a.u.) mean activations in each ROI and task condition in both groups.

	ADHD (*N* = 22)	TD (*N* = 21)	*p*		ADHD (*N* = 22)	TD (*N* = 21)	*p*
	**Left prefrontal**				**Right prefrontal**		
0 Back	.16 ± 0.82	1.78 ± 4.73	0.14[a]	0 Back	-.04 ± 1.12	.60 ± 4.25	.512[a]
1 Back	-.13 ± 1.43	.36 ± 7.00	.811[b]	1 Back	-.14 ± 1.83	4.02 ± 7.13	**.039**
2 Back	.52 ± 1.21	2.33 ± 4.15	.066[a]	2 Back	.64 ± 1.61	3.70 ± 4.15	**.004**[a]
All task	.18 ± 0.83	1.49 ± 4.23	.178[a]	All task	.15 ± 1.08	2.77 ± 4.11	**.010**[a]
	**Left frontoparietal**				**Right frontoparietal**		
0 Back	-.03 ± 0.73	1.41 ± 4.37	.154[b]	0 Back	-.11 ± 0.80	.59 ± 3.56	.636[b]
1 Back	-.15 ± 1.14	1.07 ± 5.98	.437[b]	1 Back	-.11 ± 1.03	.49 ± 6.27	.140[b]
2 Back	.59 ± 1.01	3.03 ± 4.37	**.001**[b]	2 Back	.41 ± 1.05	3.03 ± 4.87	**.003**[b]
All task	.14 ± 0.61	1.84 ± 3.75	**.010**[b]	All task	.07 ± 0.53	1.37 ± 3.95	.076[b]

Table S3b. HbR (a.u.) mean activations in each ROI and task condition in both groups.

	ADHD (*N* = 22)	TD (*N* = 21)	*p*		ADHD (*N* = 22)	TD (*N* = 21)	*p*
	**Left prefrontal**				**Right prefrontal**		
0 Back	.10 ± 1.64	-1.48 ± 4.16	.099[b]	0 Back	.34 ± 1.32	-1.87 ± 5.78	.132[b]
1 Back	.83 ± 1.94	-4.65 ± 7.58	**.004**[a]	1 Back	.68 ± 1.80	-7.20 ± 8.53	**<.001**[a]
2 Back	.23 ± 1.42	-1.99 ± 2.98	**.005**[a]	2 Back	-.11 ± 1.19	-2.94 ± 4.63	**.013**[a]
All task	.39 ± 1.21	-2.71 ± 3.93	**.002**[a]	All task	.30 ± 0.95	-4.00 ± 5.05	**<.001**[b]
	**Left frontoparietal**				**Right frontoparietal**		
0 Back	.10 ± 0.49	.04 ± 3.17	.941[b]	0 Back	.28 ± 0.83	-.24 ± 2.37	.616[b]
1 Back	.24 ± 0.97	-1.94 ± 6.25	**.023**[b]	1 Back	.08 ± 0.95	-.42 ± 5.91	.379[b]
2 Back	-.32 ± 0.91	-1.48 ± 5.74	**.029**[b]	2 Back	-.36 ± 0.72	-1.27 ± 3.39	.240[a]
All task	.01 ± 0.56	-1.13 ± 4.05	**.018**[b]	All task	-.001 ± 0.53	-.64 ± 2.95	.379[b]

Note. 0B = 0 back task condition; 1B = 1 back task condition; 2B = 2 back task condition. All task = sum of all task conditions activations. ADHD = Children with ADHD; HbO = oxyhemoglobin; HbR = deoxyhemoglobin; TD = Typically developing children. Contrast in bold is significant at alpha = .05. aStudent's t test. b Mann–Whitney U test.

Table S4a. Spearman's rho values for correlations between HbO and blood fatty acid measures in the whole sample.

	LA	AA	EPA	DHA		LA	AA	EPA	DHA
	Left prefrontal					Right prefrontal			
0 Back	−.191 [−.50; .188]	−.082 [−.425; .264]	−.029 [−.379; .275]	−.025 [−.352; .306]	0 Back	−.197 [−.511; .162]	−.046 [−.442; .325]	−.192 [−.497; .148]	.030 [−.305; .363]
1 Back	.033 [−.270; .352]	.211 [−.148; .547]	−.265 [−.536; .048]	.081 [−.291; .395]	1 Back	.133 [−.213; .425]	.272 [−.102; .550]	−.255 [−.569; .092]	.175 [−.203; .509]
2 Back	−.025 [−.385; .365]	.112 [−.260; .477]	−.078 [−.356; .223]	.116 [−.225; .431]	2 Back	−.138 [−.464; .200]	.220 [−.115; .533]	.239 [−.063; .479]	−.020 [−.339; .298]
All task	−.064 [−.386; .298]	.112 [−.240; .453]	−.221 [−.495; .095]	.037 [−.288; .361]	All task	.015 [−.326; . 315]	.198 [−.181; .518]	−.179 [−.481; .166]	.161 [−.177; .480]
	Left frontoparietal					Right frontoparietal			
0 Back	−.269 [−.533; .065]	−.131 [−.465; .228]	**−.506** ** **[−.710; . 208]**	−.023 [−.337; .320]	0 Back	−.332 * [−.626; .040]	**−.352** * **[−.625; −.011]**	**−.462** ** **[−.706; −.157]**	−.051 [−.386; .306]
1 Back	−.214 [−.515; .123]	.160 [−.205; .515]	−.326 [−.573; −.013]	.12 [−.221; .442]	1 Back	−.286 [−.565; .020]	−.055 [−.391; .298]	−.243 [−.554; .090]	.081 [−.251; .380]
2 Back	−.079 [−.408; .289]	.208 [−.130; .544]	−.071 [−.383; .268]	−.065 [−.369; .268]	2 Back	−.035 [−.385; .336]	.03 [−.283; .365]	−.012 [−.335; .354]	−.134 [−.424; .184]
All task	−.206 [−.507; .153]	.160 [−.176; .474]	**−.356** * **[−.594; −.053]**	.021 [−.299; .372]	All task	−.212 [−.516; .126]	−.054 [−.379; .275]	−.295 [−.602; .045]	−.023 [−.363; .286]

Note. HbO = Oxyhemoglobin; HbR = Deoxyhemoglobin; 0 Back = 0 back task condition; 1 Back = 1 back task condition; 2 Back = 2 back task condition. All task = sum of all task conditions activations. *$p < .05$; **$p < .01$. Values in bold are significant according to *p* value and bootstrap confidence intervals. Lower and upper limits of 95% confidence intervals from bootstrapping methodology with 1.000 resamples iteration are reported in square brackets.

Table S4b. Spearman coefficients values for correlations between HbR and blood fatty acid measures in the whole sample.

	LA	AA	EPA	DHA		LA	AA	EPA	DHA
**Left prefrontal**					**Right prefrontal**				
0 Back	.146 [−.212; .477]	.093 [−.252; .413]	−.041 [−.318; .278]	−.014 [−.341; .318]	0 Back	.038 [−.306; .345]	−.137 [−.431; .195]	−.001 [−.333; .311]	−.038 [−.395; .299]
1 Back	.001 [−.328; .335]	−.280 [−.594; .091]	.254 [−.066; .546]	**−.390** * **[−.668; −.050]**	1 Back	−.007 [−.352; .345]	−.221 [−.526; .120]	.129 [−.208; .443]	−.202 [−.493; .119]
2 Back	.012 [−.335; .336]	.095 [−.246; .414]	−.104 [−.436; .227]	−.187 [−.492; .180]	2 Back	.301 [−.035; .583]	−.119 [−.451; .214]	−.173 [−.426; .114]	−.157 [−.474; .214]
All task	.099 [−.247; .443]	−.095 [−.467; .251]	.081 [−.289; .388]	−.268 [−.580; .093]	All task	.105 [−.234; .408]	−.219 [−.550; .129]	−.022 [−.366; .302]	−.222 [−.520; .111]
**Left frontoparietal**					**Right frontoparietal**				
0 Back	.036 [−.356; . 376]	.089 [−.222; .374]	.214 [−.107; .525]	.035 [−.292; .339]	0 Back	−.083 [−.398; .252]	−.066 [−.382; .240]	**.353** * **[.046; .622]**	.125 [−.235; .442]
1 Back	.033 [−.285; .360]	−.098 [−.420; .271]	−.003 [−.317; .320]	−.212 [−.532; .170]	1 Back	.314 [.019; .571]	.119 [−.223; .428]	.071 [−.269; .400]	**−.377** * **[−.660; −.013]**
2 Back	.006 [−.310; .334]	.101 [−.218; .426]	−.165 [−.442; .135]	−.242 [−.544; .117]	2 Back	.284 [−.049; .567]	**.421** ** **[.115; .633]**	.027 [−.293; .323]	−.042 [−.378; .294]
All task	−.006 [−.343; .298]	−.002 [−.329; .333]	.030 [−.264; .347]	−.126 [−.464; .231]	All task	.304 [−.017; .567]	.290 [−.012; .541]	.119 [−.206; .431]	−.295 [−.612; .062]

Note. HbO = Oxyhemoglobin; HbR = Deoxyhemoglobin; 0 Back = 0 back task condition; 1 Back = 1 back task condition; 2 Back = 2 back task condition. All task = sum of all task conditions activations. *$p < .05$; **$p < .01$. Values in bold are significant according to p value and bootstrap confidence intervals. Lower and upper limits of 95% confidence intervals from bootstrapping methodology with 1.000 resamples iteration are reported in square brackets.
